# Memory of tolerance and induction of regulatory T cells by erythrocyte-targeted antigens

**DOI:** 10.1038/srep15907

**Published:** 2015-10-29

**Authors:** Alizée J. Grimm, Stephan Kontos, Giacomo Diaceri, Xavier Quaglia-Thermes, Jeffrey A. Hubbell

**Affiliations:** 1 Institute of Bioengineering, Ecole Polytechnique Fédérale de Lausanne, Lausanne, Switzerland; 2 Anokion SA, Ecublens, Switzerland; 3 Kanyos Bio, Inc., Cambridge, Massachusetts, USA; 4 Institute of Chemical Sciences and Engineering, Ecole Polytechnique Fédérale de Lausanne, Lausanne, Switzerland; 5 Institute for Molecular Engineering, University of Chicago, Chicago, Illinois, USA

## Abstract

New approaches based on induction of antigen-specific immunological tolerance are being explored for treatment of autoimmunity and prevention of immunity to protein drugs. Antigens associated with apoptotic debris are known to be processed tolerogenically *in vivo*. Our group is exploring an approach toward antigen-specific tolerization using erythrocyte-binding antigens, based on the premise that as the erythrocytes circulate, age and are cleared, the erythrocyte surface-bound antigen payload will be cleared tolerogenically along with the eryptotic debris. Here, we characterized the phenotypic signatures of CD8+ T cells undergoing tolerance in response to soluble and erythrocyte-targeted antigen. Signaling through programmed death-1/programmed death ligand-1 (PD-1/PD-L1), but not through cytotoxic T lymphocyte antigen 4 (CTLA4), was shown to be required for antigen-specific T cell deletion, anergy and expression of regulatory markers. Generation of CD25+FOXP3+ regulatory T cells in response to erythrocyte-targeted antigens but not soluble antigen at an equimolar dose was observed, and these cells were required for long-term maintenance of immune tolerance in both the CD4+ and CD8+ T cell compartments. Evidence of infectious tolerance was observed, in that tolerance to a one antigenic epitope was able to regulate responses to other epitopes in the same protein antigen.

Induction and reestablishment of immunological tolerance is important in situations of autoimmunity and graft-versus-host disease^1^. Advances in antigen non-specific approaches have included targeting and blocking signaling molecules required for lymphocyte activation and function, such as anti-CD3e (teplizumab)^2^ or anti-CD20 (rituximab)^3^ for inhibition of T and B cells, respectively. While beneficial effects in disease control using these therapeutics have been demonstrated, their use is limited by systemic side effects they induce due to the lack of antigen-specificity.

Several technologies engineered to establish antigen-specific immune tolerance have recently emerged^4^. Among them, soluble peptide therapy is currently used in clinic for allergy desensitization^5^ and is under clinical investigation for the treatment of celiac disease^6^ and multiple sclerosis^7^. Modeling the natural process by which tolerance to self-antigens is maintained in the periphery, DNA vaccines have been developed to induce expression of antigen by autologous healthy cells and modulate the immune system toward antigen-specific immune tolerance. Tolerability and beneficial effects of DNA vaccines have been demonstrated in phase I/II clinical trials in multiple sclerosis^8^ and type 1 diabetes^9^ (T1D) patients. In a similar way, lentivirally-induced gene expression of antigen in hepatocytes has also demonstrated tolerance in animal models of T1D^10^. Still seeking to mimic the process by which peripheral tolerance is maintained, apoptotic cells have been used as a tolerogenic carrier of antigen for induction of antigen-specific tolerance^11^. Antigen-coupled cells, which are rendered apoptotic and coupled to the antigen *ex vivo*, have shown positive outcomes in several models of autoimmunity such as T1D^12^ and multiple sclerosis^13^, in allergy^14^ and tissue transplantation^15^. Results in animal models have been encouraging, and a phase I clinical trial has shown safety and tolerability as well as signs of immunological benefits^13^.

Following the idea of using apoptotic cells as a carrier for antigens to achieve tolerogenic antigen presentation, our laboratory has been exploring targeting antigen to the surface of erythrocytes via administration of a glycophorin A-binding antigen form^16^. Due to their limited life span and inability to self-repair, billions of erythrocytes die by eryptosis, a process analogous to apoptosis, every minute and are removed from blood, representing a massive population of apoptotic cells that can be targeted for induction of immune tolerance^17^. To avoid *ex vivo* manipulation of erythrocytes, we engineered erythrocyte-binding moieties, which are recombinantly fused or chemically conjugated to the antigen, allowing binding of the antigen on erythrocytes *in situ* after intravenous injection. Treatment with erythrocyte-targeted antigens was shown to induce antigen-specific CD4+ and CD8+ T cell deletion and anergy in an ovalbumin (OVA) challenge model and a BDC2.5 T cell adoptive transfer model of T1D^16^. Our laboratory has also shown prevention of humoral immune responses to xenoantigens, such as the E. coli-derived protein drug L-asparaginase, presumably by deletion of antigen-specific CD4+ T cells that would provide help to a B cell response^18^. While antigen-specific T cell tolerance^16^ and prevention of B cell response to a protein therapeutic^18^ have been demonstrated, the molecular mechanisms implicated in the tolerance process still requires further elucidation.

In the present study, we assessed phenotypic signatures of CD8+ T cells undergoing tolerization in response to soluble versus cell-associated, here erythrocyte-bound, antigens. Among the tolerogenic molecules expressed by CD8+ T cells in response to erythrocyte-targeted antigens, signaling through PD-1, but not CTLA4 alone, was shown to be required for tolerance induction. Regulatory T cells (Tregs) were induced in response to erythrocyte-associated antigen but not free antigen at equivalent dose, regulating response to antigen challenge in both the CD4+ and CD8+ T cell compartments.

## Results

### Erythrocyte-binding antigen constructs

To evaluate the impact of cell association on the immunological response to antigens, we engineered two molecular forms of OVA, one with the full-length protein and one with only the CD8+ T cell immunodominant epitope in the context of H2-Kb. For the full-length protein, we chemically conjugated to OVA an average of three copies of the ERY1 peptide with sequence H_2_N-WMVLPWLPGTLDGGSGCRG-CONH_2_, which binds specifically to murine glycophorin A^16^. This form thus comprises both the CD4+ and CD8+ T cell epitopes of OVA, requiring proteolytic processing after internalization to free the distinct epitopes. Native OVA was used as a non-cell-associating form. For the CD8+ T cell immunodominant epitope, we formed a recombinant fusion of OVA_250-264_ with the single-chain Fv antibody fragment TER119, which binds to murine glycophorin A or an associated protein^19^. Proteolytic processing after internalization liberates the epitope OVA_257-264_, with sequence SIINFEKL^20^. Free OVA_257-264_, SIINEFKL, was used as a control.

### CD8+ T cell phenotypic signatures during tolerance induction by erythrocyte-targeted or soluble antigens

**Figure 1:**
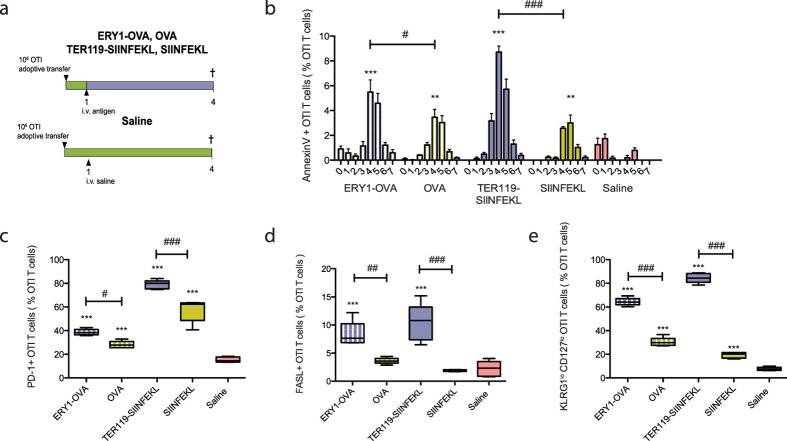
OTI T cell phenotypic markers expressions in response to soluble and erythrocyte-bound antigens. (**a**) 10^6^ CFSE-labeled OTI CD8+ T cells (CD45.1+) were adoptively transferred in C57BL/6 mice (CD45.2+) on day 0 and mice treated with erythrocyte-bound or free antigen or saline the next day. Here, the full OVA protein was used with the ERY1-OVA antigen form, compared to free OVA; and only the CD8+ T cell epitope SIINFEKL was used with the TER119-SIINFEKL antigen form, compared with free SIINFEKL peptide. Spleens were collected on day 4 for flow cytometric analysis. (**b**) AnnexinV binding per generation, (**c**) PD-1+, (**d**) FasL+ and (**e**) KLRG1^lo^ CD127^lo^ OTI T cells populations in the spleen on day 4. Data represent mean ± SD of n = 5. 1 way ANOVA *: respective to Saline group. *,#: < 0.05, **,##:< 0.01, ***,###: < 0.001..

To understand the mechanisms involved in the tolerance process to erythrocyte-associated antigens, expression of specific tolerogenic markers was measured on CD8+ T cells during induction of tolerance by erythrocyte-targeted versus soluble antigens. 10^6^ CFSE-labeled OTI T cells were adoptively transferred on day 0. Tolerance was induced by intravenous administration of soluble or erythrocyte-targeted OVA or SIINFEKL peptide. Three days later, spleens were harvested and phenotypic signatures of OTI T cells were determined by flow cytometry (Fig. 1a).

While early lymphocyte proliferation is common to both immunity and tolerance, different markers and cytokines are expressed during proliferation and dictate the fate of the cells toward effector/memory activated cells or anergy/deletion^21^. Administration of both soluble and erythrocyte-targeted antigens induced OTI T cell proliferation (Fig. S1a) and expression of tolerogenic markers such as AnnexinV-binding, PD-1 (Fig. 1b,c) and CTLA-4 (Fig. S1b). Binding of AnnexinV, indicative of apoptosis, was elevated in response to erythrocyte-targeted antigen compared to soluble antigen (Fig. 1b), and PD-1 expression was significantly higher (Fig. 1c), with CTLA4 expression being similar (Fig. S1b). In addition, a population of FasL-positive OTI T cells was observed in the group treated with erythrocyte-targeted but not soluble antigen (Fig. 1d).

Tolerance is associated with the lack of upregulation of effector features such as interferon γ (IFNγ) and granzyme B (gzmB) during T cell activation^21^. Downregulation of IFNγ was similarly observed in response to soluble and erythrocyte-bound antigen (Fig. S1c) and only in response to free OVA was an increase in gzmB production observed (Fig. S1d); erythrocyte association of OVA completely abrogated this response. Ly6C, which is associated with cytotoxic activity, was also downregulated in response to both erythrocyte-targeted and soluble antigens (Fig. S1e). KLRG1 expression, related to terminally differentiated CD8+ T cells and senescence^22^,^23^, and expression of CD127 (IL-7R), which is associated with memory phenotypes and downregulation of which has been linked to deletional tolerance, were also measured^21^. Expression of both CD127 and KLRG1 were significantly lower in response to erythrocyte-targeted versus soluble antigens (Fig. S1f–g), and OTI T cells were in majority KLRG1^low^CD127^low^ in response to erythrocyte-targeted antigens while being mostly KLRG1^low^CD127^high^ in response to soluble antigens (Fig. 1e). Finally, no expression of TRAIL (Fig. S1h), related to marking for deletion, or change in CD62L/CD44 expression (Fig. S1i), related to T cell homing, were observed in response to erythrocyte-targeted and soluble antigens. Gating strategies are depicted in Fig. S2 and Fig. S3.

Taken together, these results show that erythrocyte association of antigen leads to more extensive CD8+ T cell cross-priming, but that the phenotype of the proliferating T cells bears characteristics of cross-tolerization more than effector cross-priming.

### Co-blockade of PD-1 and CTLA4 signaling abrogates antigen-specific deletion in response to erythrocyte-targeted antigens

**Figure 2:**
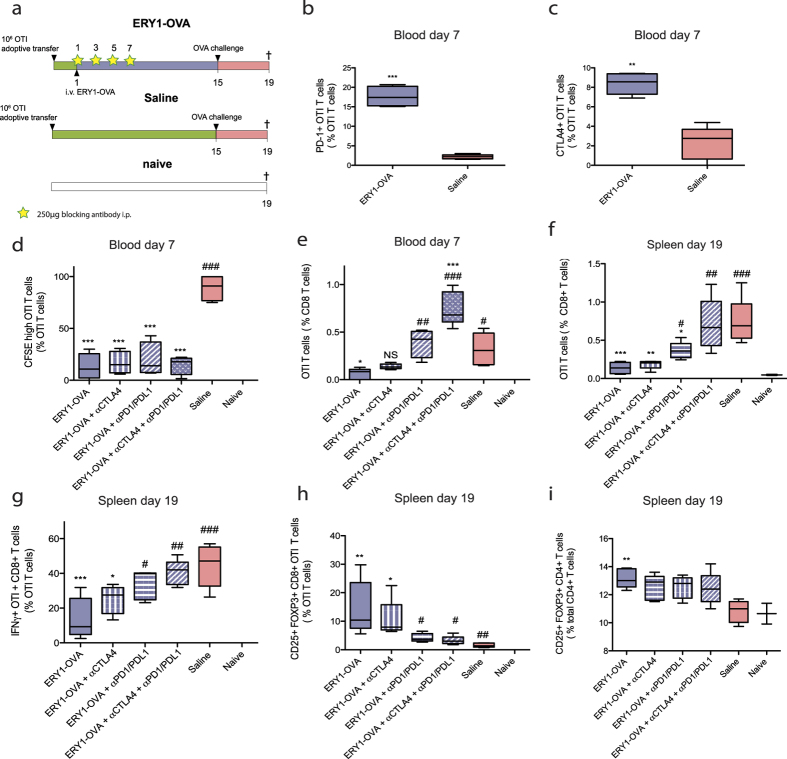
Co-blockade of PD-1/PD-L1 and CTLA4 signaling abrogates antigen-specific T cell deletion by erythrocyte-targeted antigens. (**a**) 10^6^ CFSE-labeled OTI CD8+ T cells (CD45.1+) were adoptively transferred in C57BL/6 mice (CD45.2+) on day 0. The next day, mice were treated i.v. with ERY1-OVA to induce proliferation and deletion of OTI T cells. 250 μg each of αPD-1 and αPD-L1 administered together, or 250 μg αCTLA4, or 250 μg each of all three antibodies were administered i.p as indicated during the period of putative tolerization. Finally, mice were challenged i.d with OVA + LPS on day 15 and organs were harvested 4 days later for flow cytometric analysis. Measured in the blood 6 days after injection of 10 μg ERY1-OVA or saline: (**b**) proliferation of OTI T cells, **(c**) PD-1+ and (**d**) CTLA4+ OTI T cell populations, and (**e**) deletion of OTI T cells. Measured in the spleen on day 19, 4 days after antigen challenge: (**f**) OTI T cell response to challenge, (**g**) IFNγ+ OTI T cells from spleen after 6 hours *in vitro* restimulation with SIINFEKL peptide, frequency of (**h**) CD25+FOXP3+CD8+ OTI T cells and (**i**) CD25+FOXP3+CD4+ endogenous (CD45.2+) T cells. Data represent mean ± SD of n = 5. 1 way ANOVA *: respective to Saline group, #: respective to ERY1-OVA group. *, #: < 0.05, **, ##: < 0.01, ***, ###: < 0.001.

As PD-1 and CTLA4 are upregulated on proliferating OTI T cells in response to erythrocyte-targeted antigens (PD-1 differentially in response to erythrocyte binding, CTLA4 not) and are commonly associated with immune tolerance^22^, we evaluated the requirement of these signaling molecules for induction of antigen-specific T cell deletion, anergy and differentiation into Tregs in response to erythrocyte-targeted OVA. 10^6^ CFSE-labeled OTI T cells were adoptively transferred on day 0. One day later, mice were injected intravenously with ERY1-OVA to induce OTI T cell proliferation and deletion. PD-1- and CTLA4-induced signaling were prevented during the tolerization phase by i.p. administration of 250 μg blocking antibodies αPD-1 plus αPD-L1 or αCTLA4 alone, or the blocking antibodies combined. Mice were challenged on day 15 with OVA + LPS i.d. and sacrificed on day 19 for organ collection, *in vitro* restimulation and flow cytometric analysis (Fig. 2a), based on the premise that a lack of response to challenge is indicative of tolerance. Proliferation and deletion of OTI T cells were assessed in blood on day 7 and in the spleen and draining lymph nodes (dLN) on day 19, four days following antigen challenge.

Significant increases in PD-1+ and CTLA4+ OTI T cell populations were observed in response to ERY1-OVA compared to saline control (Fig. 2b,c). Proliferation of OTI T cells in response to ERY1-OVA was not affected by blockade of PD-1/PD-L1 or CTLA4 alone, or by co-blockade of the two signaling pathways (Fig. 2d). While no effects of blocking CTLA4 or PD-1/PD-L1 signaling were observed regarding proliferation of the OTI T cells (Fig. 2d), blockade of PD-1/PD-L1 significantly reduced subsequent deletion of proliferated OTI T cells (Fig. 2e), suggesting a role of PD-1/PD-L1 signaling in induction of apoptosis of CD8+ T cells during the tolerance process induced by erythrocyte-associated antigens. No effect was observed when CTLA4 was blocked alone, however co-blockade of PD-1/PD-L1 and CTLA4 lead to a stronger effect on tolerance induction compared to blockade of PD-1/PD-L1 alone, as it completely prevented OTI T cell deletion as measured in the spleen four days after antigen challenge (Fig. 2f). In addition, after blockade of PD-1/PD-L1 and CTLA4 signaling, remaining OTI T cells were able to respond to *in vitro* antigen restimulation, as measured by production of IFNγ in response to restimulation with SIINFEKL for 6 hours, whereas this ability was limited after treatment of ERY1-OVA without blockade (Fig. 2g).

The requirement of PD-1/PD-L1 and CTLA4 signaling for induction of CD25+FOXP3+ OTI and endogenous Tregs by erythrocyte-targeted antigens was assessed. An average of 35 ± 10% of the remaining OTI T cells in ERY1-OVA treated group were CD25+FOXP3+ at day 19 in the spleen, compared to 6.3 ± 0.6% in the control saline group (Fig. 2h). Blockade of PD-1/PD-L1 and co-blockade of PD-1/PD-L1 with CTLA4 abrogated FOXP3 expression, while no significant effect of blocking CTLA4 alone was observed. An increase in CD25+FOXP3+CD4+ endogenous Tregs (CD45.2+) was observed in response to ERY1-OVA (Fig. 2i). Similar results were obtained in characterization of responses in dLNs (Fig. S4). The absolute numbers of OTI T cells analyzed are depicted in Fig. S4.

Taken together, these results show that the tolerogenic effect of erythrocyte-associated antigen requires stimulation the PD-1/PD-L1 axis, and that CTLA4 contributes, although to a lesser extent. This was true when considering T cell deletion, T cell response to antigen challenge, and generation of Tregs.

### Establishment of memory of tolerance and induction of regulatory T cells

**Figure 3:**
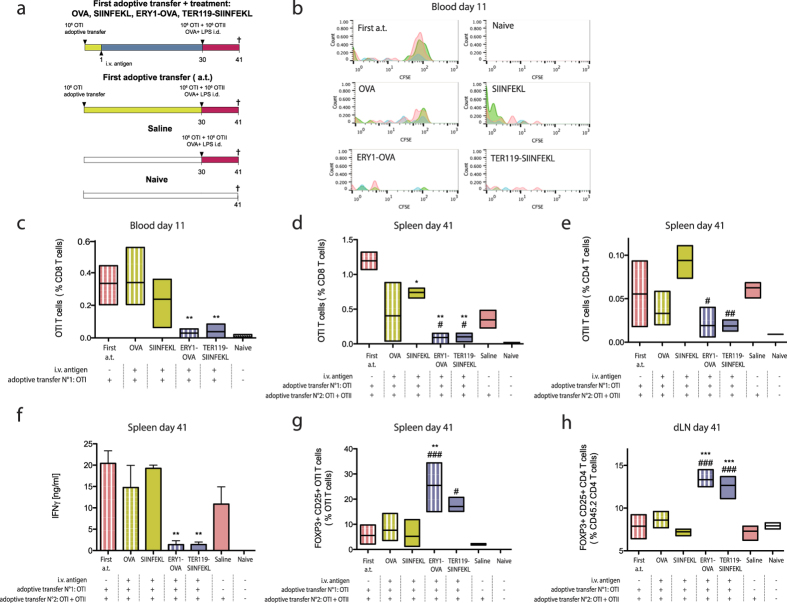
Memory of tolerance and induction of CD25+FOXP3+ OTI and endogenous CD4+ Tregs cells after treatment with erythrocyte-targeted antigens. (**a**) 10^6^ CFSE-labeled OTI CD8+ T cells (CD45.1+) were adoptively transferred on day 0 in C57BL/6 mice (CD45.2+) and mice treated with erythrocyte-bound or free antigen or saline the next day. Here, the full OVA was used with the ERY1-OVA antigen form, compared to free OVA; and only the CD8+ T cell epitope SIINFEKL was used with the TER119-SIINFEKL antigen form, compared with free SIINFEKL peptide. One month later, a second adoptive transfer of OTI and OTII T cells, with OVA+LPS i.d. challenge rather than additional molecular tolerization, was performed to assess long-term memory of tolerance. (**b**) Proliferation of OTI T cells and (**c**) OTI T cell population on day 11 in the blood. (**d**) CD8+ OTI and (**e**) CD4+ OTII T cell populations in the spleen on day 41, 10 days after the second adoptive transfer and antigen challenge. (**f**) IFNγ production after 3 days *in vitro* restimulation of splenocytes with OVA, measured by ELISA. (**g**) CD25+FOXP3+ OTI T cells in the spleen and (**h**) CD25+FOXP3+CD45.2+ (endogenous) CD4+ T cells in the draining lymph nodes on day 41. Data represent mean  ± SD of n = 3. 1 way ANOVA *: respective to first adoptive transfer group, #: respective to Saline group. *, #: < 0.05, **, ##: < 0.01, ***, ###: <0.001. Study representative of two independent experiments.

Treatment with erythrocyte-targeted antigens was previously shown to induce antigen-specific CD4+ and CD8+ T cell deletion and anergy in T cell adoptive transfer studies^16^. To determine if the established tolerance has a form of memory, we performed a similar study in which 10^6^ OTI CD8+ T cells were adoptively transferred on day 0, and soluble or erythrocyte-bound antigen were administered i.v. one day later to induce antigen-specific T cell tolerance (Fig. 3a). Consistent with results described above, treatment with erythrocyte-targeted antigens lead to the proliferation (Fig. 3b) and deletion (Fig. 3c) of adoptively transferred OTI T cells, as measured in blood on day 11. To assess memory of tolerance, a second set of ovalbumin-specific T cells was adoptively transferred one month later, namely 10^6^ OTI CD8+ T cells plus 10^6^ OTII CD4+ T cells, and mice were challenged i.d with OVA + LPS. Evidence of abrogation of expansion of the second set of adoptively transferred cells, which were challenged with antigen without additional molecular tolerization, would be taken as functional evidence of regulation induced by the previous tolerogenic dose. In the groups that received erythrocyte-targeted antigens, tolerance was shown to be maintained one month after treatment, as the second set of adoptively transferred T cells was deleted in the groups treated with erythrocyte-targeted antigens but not soluble antigens in both the CD8+ and the CD4+ T cell compartments (Fig. 3d,e). Indeed, treatment with ERY1-OVA and TER119-SIINFEKL not only induced proliferation and deletion of the first adoptively transferred T cells (Fig. 3b,c), but in addition led to the suppression of the second adoptively transferred OTI and OTII T cells and a global reduction in the magnitude of IFNγ produced by splenocytes after *in vitro* restimulation with OVA (Fig. 3f). By all measures, treatment with ERY1-OVA and TER119-SIINFEKL were able to induce functional regulation, but free OVA and free SIINFEKL at the same molar doses were not able to do so. Interestingly, tolerization with TER119-SIINFEKL, comprising only the CD8+ T cell epitope, was able to regulate OVA-specific CD4+ T cells after challenge with the full OVA protein adjuvanted with LPS. Similar results were obtained in the dLN (Fig. S5) and absolute numbers of OTI and OTII T cells analyzed are depicted in Fig. S5.

Tregs being central for induction of robust long-term tolerance, frequencies of CD25+FOXP3+ CD8+ and CD4+ Tregs were assessed on day 41, 10 days after antigen challenge. Interestingly, larger fractions of CD25+FOXP3+OTI T cells in both the spleen and dLN were measured in response to erythrocyte-targeted antigens (Fig. 3g). An increase in the frequency of CD25+FOXP3+CD4+ endogenous Tregs (CD45.2+) was also observed in response to erythrocyte-targeted antigens in dLN (Fig. 3h), with no difference observed in the spleen. No differences in frequency of endogenous CD25+FOXP3+CD8+ Tregs (CD45.2+) were observed in the spleen and dLN (Fig. S5f).

These results thus demonstrate that erythrocyte-associated antigen, contrasted with free antigen at the same molar dose, induces endogenous CD4+ regulatory T cells, expression of FOXP3 in adoptively transferred OTI T cells, and dampens a response to antigen challenge to T cells that have not been exposed to the tolerogenic dose, thus providing both molecular and functional evidence of memory of tolerance. Interestingly, tolerance induced by a CD8+ T cell epitope (TER119-SIINFEKL) was able to regulate a response to challenge to the whole antigen (OVA, comprising both CD4+ and CD8+ T cell epitopes) in both CD4+ and CD8+ T cell compartments, further supporting the premise of functional regulation.

### Depletion of CD25+ T cells abrogates the tolerance state

To assess the requirement of regulatory T cells for the maintenance of tolerance, Tregs were depleted using a depletion αCD25 antibody prior to the second adoptive transfer of OTI T cells (Fig. S6a). 250 μg of αCD25 were injected i.p. on day 23 and 30 to deplete all CD25+ T cells. CD25+ T cell depletion was confirmed by flow cytometric measurement of circulating blood cells (Fig. S6b). Depletion of CD25+ T cells abrogated tolerance established by erythrocyte-targeted antigens, and second adoptively transferred OTI T cells were not deleted in response to earlier ERY1-OVA treatment if mice were later exposed to αCD25 antibody (Fig. S6c). While these data would suggest the requirement of CD25+ Tregs in the maintenance of tolerance, it must be mentioned that the control untolerized group was also significantly affected by αCD25. Indeed, a significant increase in the frequency of OTI T cells in the spleen and dLN was observed when mice were previously depleted of their CD25+ T cells (Fig. S6c).

### Memory of tolerance is induced by endogenous regulation and does not require CD25+FOXP3+ OTI T cells

**Figure 4:**
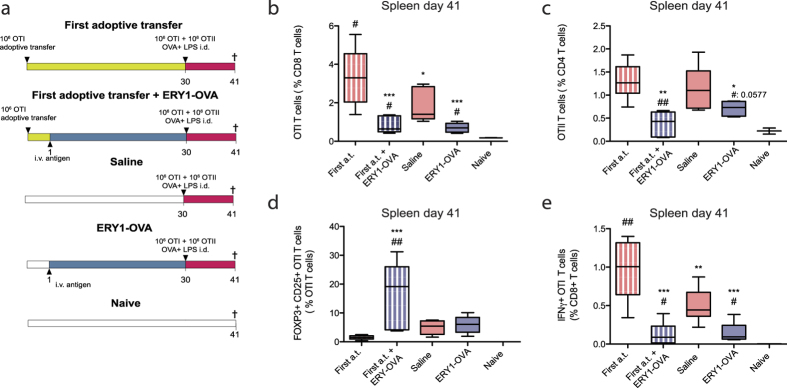
Memory of tolerance can be induced by exclusively endogenous T cell populations. (**a**) 10^6^ OTI CD8+ T cells (CD45.1+) were adoptively transferred into C57BL/6 mice (CD45.2+). The next day, mice were treated i.v. with ERY1-OVA to induce proliferation and deletion of OTI T cells. One month later, adoptive transfer of OTI and OTII T cells, with OVA+LPS i.d. challenge rather than additional molecular tolerization, was performed to assess long-term memory of tolerance. (**b**) OTI CD8+ T cell, (**c**) OTII CD4+ T cell and (**d**) CD25+FOXP3+ OTI T cell populations in the spleen on day 41. (**e**) IFNγ+ OTI T cells after 6 hours *in vitro* restimulation with SIINFEKL. Data represent mean ± SD of n = 5. 1 way ANOVA *: respective to first a.t. group, #: respective to Saline group. *, #: < 0.05, **, ##: < 0.01, ***, ##:  < 0.001.

As shown above, after adoptive transfer of OTI T cells, treatment with erythrocyte-targeted antigen lead to an increased frequency of CD25+FOXP3+ OTI T cells, which correlated with the establishment of memory of tolerance. To understand if induced CD25+FOXP3+OTI T cells are required for regulation of post-tolerization adoptively transferred OTI and OTII T cells, or if instead regulation by endogenous T cell populations is sufficient, a study was carried out in which mice did or did not receive the initial OTI T cell adoptive transfer. Mice were adoptively transferred with 10^6^ OTI T cells or received saline on day 0. On day 1, mice were treated with ERY1-OVA or saline. To assess long-term tolerance, 10^6^ OTI CD8+ T cells plus 10^6^ OTII CD4+ T cells were adoptively transferred on day 30 and mice challenged i.d. with OVA + LPS (Fig. 4a).

Treatment with ERY1-OVA induces regulation of subsequently adoptively transferred OTI and OTII T cells (Fig. 4b,c), independently of the initial adoptive transfer of OTI T cells, with no statistical difference in number of OTI T cells remaining non-deleted between the groups receiving the pre-tolerogenesis adoptive transfer or not. A significant increase in the CD25+FOXP3+OTI T cell population was detected only in the ERY1-OVA group that received the first OTI adoptive transfer, suggesting that these CD25+FOXP3+OTI T cells emerge from the initial adoptive transfer and not the second adoptively transferred OTI T cells (Fig. 4e). IFNγ+ OTI T cell population after 6 hours *in vitro* restimulation with SIINFEKL was also significantly reduced in both ERY1-OVA treated groups, i.e. with and without the initial OTI T cell adoptive transfer, with no difference with and without the pre-tolerogenesis adoptive transfer (Fig. 4f). Similar results were obtained in dLN (Fig. S7).

These results demonstrate that endogenous OVA-reactive T cell populations are sufficient to induce memory of tolerance capable of down-regulating even a strongly adjuvanted antigen challenge.

## Discussion

Early signaling received after recognition of an antigen by the TCR dictates the fate of the corresponding lymphocyte toward activation into an effector/memory phenotype or toward tolerance^24^. In the absence of costimulation or in the presence of co-inhibitory signals, lymphocytes become unresponsive, die by apoptosis or are differentiated into Tregs^25^. Tregs are critical for maintenance of tolerance and are able to suppress autoreactive T cells in the periphery in a direct manner, such as by IL-2 deprivation or production of cytotoxic cytokines, or indirectly by modulating dendritic cells (DCs) toward tolerogenic presentation of the antigen^26^. Tregs are recognized by the expression of the transcription factor FOXP3 and high levels of the surface protein CD25, the receptor for IL-2^27^. The role of Tregs in establishment and maintenance of tolerance is emphasized by the fact that humans having mutations in the gene encoding FOXP3 rapidly develop autoimmunity^28^. Furthermore, reduced levels of CD4+ Tregs cells have been described in several autoimmune diseases^27^. While CD25+FOXP3+CD4+ Tregs have been regarded as the main player in prevention of autoimmunity, the requirement of CD8+ Tregs cells for maintenance of peripheral tolerance has recently gained interest, where high levels of CD25+FOXP3+CD8+ Tregs cells have been associated with remission of multiple sclerosis^29^ and reduced graft-versus-host disease response^30^.

In this study, we assessed the phenotypic signatures of T cells undergoing tolerance in response to soluble or erythrocyte-associated antigens. While sharing some common features, such as expression the anergic markers PD-1, CTLA4 and increase in AnnexinV-binding during proliferation as well as downregulation of the cytotoxic marker Ly6C and production of pro-inflammatory cytokines such as IFNγ and gzmB, the magnitude of these changes differed between the two antigen delivery modalities (Fig. 1). Indeed, expression levels of PD-1 and AnnexinV-binding were significantly elevated when antigen was targeted to the erythrocyte surface compared to its equivalent soluble format (Fig. 1b,c). Interestingly, in response to erythrocyte-targeted antigens a population of FasL+ OTI T cells was shown to be induced (Fig. 1d). FasL has been shown to be expressed by CD8+ Tregs cells and implicated in subsequent maintenance of tolerance through CD4+ T cell deletion^31^. As no CD25+FOXP3+OTI T cells were detected at this early time point, further studies would be required to assess if expression of FasL is maintained and if these cells eventually differentiate into functional Tregs. Finally, in response to erythrocyte-targeted antigens, proliferated OTI T cells were mostly CD127^low^ KLRG1^low^, while being CD127^high^ KLRG1^low^ in response to soluble antigens (Fig. 1e). Downregulation of CD127/IL-7 Rα is observed during CD8+ T cell deletional tolerance and exhaustion where loss of IL-7 signaling eventually results in T cell apoptosis^21^,^22^,^32^. In the context of tolerance, low expression of CD127 has been used as a marker for Tregs^33^, and supplementary studies would be required to determine if low expression is maintained on OTI T cells after erythrocyte-associated antigen delivery.

Among the various inhibitory receptors associated with tolerance, the role of PD-1 and CTLA4 in induction of peripheral tolerance and limiting autoimmunity is well established^34^ and is known to play a role in tolerance to antigens associated with apoptotic cell debris^35^,^12^. Due to their implication in maintenance of tolerance and their upregulation during proliferation in response to erythrocyte-associated antigens (Fig. 2b,c), we assessed the requirement of these signals for tolerance induction. Blocking CTLA4 or PD-1/PD-L1 signaling did not impact the initial proliferation of OTI T cells in response to erythrocyte-targeted antigen (Fig. 2d). Our results indicate that CTLA4 signaling is not required for induction of tolerance by erythrocyte-targeted antigens, as blocking it using an αCTLA4 antibody did not diminish induction of tolerance. As CTLA4 is associated with effector function of Tregs^26^, it would be of interest to understand if CTLA4 signaling is instead required for maintenance of tolerance by induced regulatory T cells. While no effect was observed after blockade of CTLA4, PD-1/PD-L1 signaling was shown to be required for induction of antigen-specific T cell deletion, anergy and differentiation into Tregs (Fig. 2e–g). Interestingly, complete prevention of OTI T cell deletion required co-blockade of PD-1/PD-L1 and CTLA4 (Fig. 2e–g). While early molecular events differ between signaling induced by CTLA4 and PD-1, activation of both receptors converges with the inhibition of AKT phosphorylation, which is required for T cell activation, cytokine production and survival^34^,^36^. It is possible that signaling through one of these receptors can compensate the lack of signaling of the other, explaining why both signaling mechanisms needed to be blocked to completely abrogate induction of tolerance.

Establishment of sustained tolerance requires induction of regulatory T cells. We thus assessed long-term tolerance and generation of CD25+FOXP3+OTI T cells as well as endogenous CD8+ and CD4+ Tregs cells in response to both soluble OVA or its CD8+ T cell epitope SIINFEKL, compared to that induced by their erythrocyte-targeted forms. Soluble SIINFEKL and erythrocyte-targeted SIINFEKL and OVA induced potent proliferation (Fig. 3b), although only the erythrocyte-targeted antigens induced strong deletion of OTI T cells (Fig. 3c). Memory of tolerance seemed to be established only when antigens were targeted to erythrocytes (Fig. 3d–f). Indeed, after infusion of a second set of OVA-specific CD4+ and CD8+ T cells, only mice that were previously treated with erythrocyte-targeted antigens were able to suppress this second set of cells and maintain tolerance (Fig. 3d,e). The half-lives of the erythrocyte-antigen constructs are about 13 hr for ERY1-OVA protein conjugate and 34 hr for TER119-SIINFEKL fusion protein; as such, the erythrocyte-targeted antigens are not detectable in the blood after 10 days and in the spleen and liver after 12 days (Fig. S8). It is thus unlikely that the effect observed in the groups treated with erythrocyte-targeted antigens, i.e. long-term memory of tolerance, is due to the presence of remaining antigen in circulation one month after injection. Rather, maintenance of tolerance correlated with induction of CD25+FOXP3+OTI and endogenous CD25+FOXP3+CD4+ Tregs (Fig. 3g,h). The ability of OTI T cells to express the surface marker CD25 and the transcription factor FOXP3 and exert suppressive activity *in vitro* and *in vivo* has been suggested by others^37^,^38^. Still, in the present study, CD25+FOXP3+OTI were not required for maintenance of tolerance, as long term tolerance was also observed when mice treated with ERY1-OVA did not receive the initial adoptive transfer of OTI T cells (Fig. 4). Thus endogenously generated regulatory T cells are capable of providing tolerance to a subsequent adoptive transfer of antigen-specific T cells and adjuvanted antigen challenge. Using an αCD25 depleting antibody further suggested that endogenous CD25+ Tregs are required for maintenance of tolerance, as no significant deletion of the subsequent set of OTI T cells was observed in response to ERY1-OVA when mice were depleted of their CD25+ T cells (Fig. S6). Such abrogation of tolerance after depletion of CD25+ T cells has also been documented regarding apoptotic splenocyte-coupled antigens, where CD25+ Tregs were shown to be dispensable for tolerance induction but required for its long-term maintenance^35^.

Interestingly, in response to both erythrocyte-targeted antigens presenting the full OVA sequence, i.e. ERY1-OVA, and only the MHC-I epitope SIINFEKL, i.e. TER119-SIINFEKL, not only OTI CD8+ T cells were deleted during the post-tolerization adoptive transfer and adjuvanted antigen challenge, but also OTII CD4+ T cells (Fig. 3e). As no ovalbumin-specific OTII T cell epitope is present in the TER119-SIINFEKL construct, this result indicates a form of infectious tolerance by which Tregs specific for the SIINFEKL epitope can drive tolerance toward other OVA epitopes in the face of challenge with the whole OVA protein. As a mechanism for maintenance of peripheral tolerance, regulatory T cells can affect autoreactive T cells in an indirect manner through downregulation of costimulation on DCs presenting the antigen^26^. By these means, Tregs specific for one epitope can establish tolerance to the whole antigen. Alternatively, CD8+ Tregs have been shown to be able to directly kill CD4+ T cells in an antigen-specific manner, by induction of apoptosis through Fas-FasL signaling^31^. Finally, infectious tolerance by Tregs has been suggested by others in studies carried out in the BDC2.5 T cell (which recognize the islet beta cell antigen chromogranin A) adoptive transfer model, where transferring BDC2.5 Tregs could prevent destruction of pancreatic islets in mice that had insulin-specific T cells^39^, referred to as bystander suppression; or in an OTI adoptive transfer model where CD25+FOXP3+OTI T cells were able to suppress CD4+ T cells and prevent OVA-expressing cardiac allograft rejection in an antigen-specific manner^38^.

In this study, we were interested in the mechanisms of immunological tolerance to cell-associated antigens, here erythrocyte-associated antigens attained through antigens with engineered affinity for glycophorin A. Interestingly, studies with transgenic erythrocytes have shown a state of immunological non-responsiveness to xenoantigens expressed on the surfaces of erythrocytes transfused under non-inflammatory conditions^40^. The concept of our therapeutic design is that as erythrocytes circulate, age and are cleared, their antigenic payload will be cleared and subjected to tolerogenic antigen presentation. We had previously shown antigen-specific deletion^16^ and ability to resist repeated antigen challenge at the humoral level^18^, but we did not know the extent to which regulatory T cells are involved and to which immunological memory of a tolerogenic dose exists. Here, we show that CD25+FOXP3+ Tregs are indeed induced, in both the CD4+ and CD8+ T cell compartments, and we show that response of both CD4+ and CD8+ T cells to an antigen challenge can be ameliorated by a previous tolerizing dose, thus demonstrating both molecular and functional evidence of immune regulation. These effects were not observed with antigens not associated to erythrocytes when evaluated at the same doses. Our results also demonstrated infectious tolerance, in that memory to a tolerogenic dose of one epitope was able to ameliorate response to another epitope in the same antigenic protein challenge. While these data suggest a role for Tregs in tolerance induced by erythrocyte-targeted antigens, the role of B cells as activate regulators still needs to be investigated. These results form the basis for continued exploration of erythrocyte targeting in prophylactic and therapeutic tolerization.

## Methods

### Animals

All studies were carried out in accordance with procedures approved by the Swiss Veterinary Authority and the EPFL Centre d’Application du Vivant. Male CD45.2+ C57BL/6 mice (Harlan) aged 8–12 wk were used for *in vivo* studies. To generate CD45.1+ OTI and OTII mice, C57BL/6-Tg(TcraTcrb) 1100Mjb (OTI) and C57BL/6-Tg(TcraTcrb)425Cbn/Crl (OTII) mice (Jackson Laboratories) were crossed with C57BL/6-Ly5.1 (Charles River) and bred in specific pathogen-free (SPF) conditions at the Ecole Polytechnique Fédérale Lausanne Animal Facility.

### TER119-Antigen

The TER119 scFv fused to SIINFEKL peptide antibody fragment was created as described elsewhere^16^ and inserted in a pSectagA expression vector (Life Technologies) for expression in HEK293E cells under serum-free conditions with 3.75 mM valproic acid (Sigma–Aldrich) for 7 d. Proteins were purified from supernatant using immobilized metal ion affinity chromatography on an Akta FPLC system and by size-exclusion using a Superdex 75 column (GE Healthcare). After purification, purity was assessed by SDS/PAGE, concentration determined by nanodrop and endotoxin levels measured using THP-1 × Blue cells (InvivoGen). Final products were sterile-filtered and stored at −80 °C.

### ERY1 chemical conjugation

10 equivalents of ERY1 peptide (H_2_N-WMVLPWLPGTLDGGSG-CRG-CONH_2_) were chemically conjugated to OVA using a sulfosuccinimidyl-4-(*N*-maleimidomethyl) cyclohexane-1-carboxylate linker (Thermo Scientific). In summary, SMCC was dissolved in dimethylformamide (DMF) and 10 molar equivalents were reacted with endotoxin-free ovalbumin (OVA; Hyglos GmbH) at a concentration of 5 mg/mL in PBS (Dulbecco’s, Sigma-Aldrich) for 2 hr at RT. Unreacted SMCC was removed from the solution using a Zeba desalting column (Thermo Scientific). The OVA-SMCC product was subsequently reacted with 10 molar equivalents of ERY1 peptide dissolved in 3 M GndHCl for 2 hr in PBS. The final product was loaded in a Zeba desalting column to remove unreacted ERY1, sterile-filtered and stored at −20 °C.

### OTI and OTII T cell adoptive transfer

CD8+/CD4+ T cells from spleen and dLN of CD45.1+ OTI/OTII mice were isolated using a CD8+/CD4+ magnetic bead negative selection kit (Miltenyi Biotec), following the manufacturer’s instructions. CD8+ OTI cells and CD4+ OTII T cells were resuspended in PBS (Dulbecco’s PBS) and labeled with 1 μM CFSE (Invitrogen) for 6 min at RT, and quenching was performed by adding an equal volume of Iscove’s modified Dulbecco’s medium (IMDM) with 10%(vol/vol) FBS (Gibco). For adoptive transfer, 100 μL of cell suspension at a concentration of 10^7^ cells/mL in IMDM was injected intravenously in the tail vein of CD45.2+ C57BL/6 mice.

### PD-1/PD-L1 and CTLA4 blockade

10^6^ CFSE-labeled CD8+ OTI cells were injected i.v. into the tail vein of recipient CD45.2+ C57BL/6 mice. 10 μg ERY1-OVA (223 pmol) were administered in a 100 μL volume of saline solution 24 hr following adoptive transfer. On days 1, 3, 5 and 7, 250 μg of blocking antibodies αPD-1 (clone RMP1-14), αPD-L1 (clone 10 F.9G2) or αCTLA4 (clone 9D9) (BioXcell) were injected intra-peritoneally. 15 days following adoptive transfer, mice were challenged with 10 μg of OVA and 50 ng of ultrapure E. coli LPS (InvivoGen) in 25 μL of saline, injected intradermally in the top of each footpad. Mice were euthanized on day 19 and the spleen and dLN were harvested for *in vitro* restimulation and flow cytometry staining. Spleens and dLN cells were restimulated *in vitro* in the presence of 1 mg/mL OVA or 1 μg/mL SIINFEKL peptide (Genscript) for 6 h. After 3 h of *in vitro* restimulation, Brefeldin-A (5 μg/mL; Sigma) was added and intracellular cytokine expression was assessed by flow cytometry analysis. Restimulation was also carried out over 3 days for measurement of secreted cytokines by ELISA using the IFNγ Ready-Set-Go Kit (eBioscience).

### Memory of tolerance and Treg studies

10^6^ CFSE-labeled CD8+ OTI cells were injected i.v. into the tail vein of recipient CD45.2+ C57BL/6 mice. 223 pmol of ERY1-OVA or OVA was injected in a 100 μL volume of saline solution 24 h following adoptive transfer. Blood was taken on day 11 to measure T cell proliferation and deletion. One month following adoptive transfer, mice were challenged with 10 μg of OVA and 50 ng of ultrapure E. coli LPS (InvivoGen) in 25 μL of saline injected intradermally in the top of each footpad. On the same day, right prior OVA + LPS challenge, mice received 10^6^ CFSE-labeled CD8+ OTI cells and 10^6^ CFSE-labeled CD4+ OTII cells. Mice were euthanized on day 41 and spleen and dLN were harvested for *in vitro* restimulation and flow cytometry staining. Spleens and dLN cells were restimulated *in vitro* in the presence of 1 mg/mL OVA or 1 μg/mL SIINFEKL peptide (Genscript) for 6 h. After 3 h of *in vitro* restimulation, Brefeldin-A (5 μg/mL; Sigma) was added and intracellular cytokine production was assessed by flow cytometry analysis. Tregs were depleted by two intraperitoneal injections on day 23 and 30 of 250 μg αCD25 antibody (clone PC61, BioXcell).

### Flow cytometry

Flow cytometry measurements were performed using a CyAn ADP Analyzer (Beckman Coulter) and data were analyzed using version 9.8.2 of FlowJo software. Viable cells were detected by LIVE/DEAD (L/D) Fixable Aqua stain (Invitrogen) and the following antibodies were used for surface and intracellular staining: CD3e Pacific Blue, CD4 FITC, CD8 APC-Cy7, CD45.1 Pe-Cy7, CD25 PE, PD-1 PE, CTLA4 APC, IFNγ APC, FOXP3 PerCP-Cy5.5 (eBioscience). For staining of blood and splenocytes, cells were exposed for 5 min at RT to 0.155 NH_4_Cl to lyse erythrocytes. The following staining steps were performed on ice. Cells were washed with PBS, stained for 15 min with Aqua L/D stain, resuspended in PBS + 2% FBS for surface staining for 15 min and finally fixed for 15 min in PBS + 2% PFA. IFNγ intracellular staining was performed in PBS + 2% FBS supplemented with 0.5% Saponin. Foxp3 Transcription Factor Fixation/Permeabilization Concentrate and Diluent kit (ebioscience) was used for FOXP3 staining.

### Clearance of ovalbumin from liver and spleen

223 pmoles ERY1-OVA, OVA or saline were injected intravenously in mice on day 0. Organs were collected at specific time points and deep frozen in liquid nitrogen. Proteins were extracted from spleen and liver using T-per tissue protein extraction reagent (ThermoFisher Scientific) containing a protease inhibitor cocktail (Sigma-Aldrich). Primary rabbit anti-OVA antibody (Serotec 0220-1682 G) and secondary goat anti-rabbit HRP (Biorad) were used to detect ovalbumin from spleen and liver protein extracts by Western blotting. Primary mouse anti-β actin antibody (Abcam) was used as assay concentration control.

### Data Analysis

Graphing and statistical analyses of worked-up data were performed using Prism (v5; GraphPad). All flow cytometry data were analyzed using FlowJo (v9.8.2; TreeStar). 1 Way ANOVA with Bonferonni posttest, α = 0.05, was used for interpreting flow cytometry and ELISA data (***P < 0.001; **P < 0.01; *P < 0.05).

## Additional Information

**How to cite this article**: Grimm, A. J. *et al.* Memory of tolerance and induction of regulatory T cells by erythrocyte-targeted antigens. *Sci. Rep.* **5**, 15907; doi: 10.1038/srep15907 (2015).

## Supplementary Material

Supplementary Information
