# Supplementary material for: Memory of tolerance and induction of regulatory T cells by erythrocyte-targeted antigens
Source: Sci Rep. 2015 Oct 29;5:15907. doi: 10.1038/srep15907 (PMC4625129; doi:10.1038/srep15907)
Supplement: Supplementary Information [file srep15907-s1.pdf]

Supplementary information

Memory of tolerance and induction of regulatory T cells by erythrocyte-targeted antigens

Alizée J. Grimm[1], Stephan Kontos[1,2,3], Giacomo Diaceri[1], Xavier Quaglia-Thermes[1], Jeffrey A. Hubbell[1,2,3,4,5,*]

[1] Institute of Bioengineering, Ecole Polytechnique Fédérale de Lausanne, Lausanne, Switzerland

[2] Anokion SA, Ecublens, Switzerland

[3] Kanyos Bio, Inc., Cambridge, Massachusetts, USA

[4] Institute of Chemical Sciences and Engineering, Ecole Polytechnique Fédérale de Lausanne, Lausanne, Switzerland

[5] Institute for Molecular Engineering, University of Chicago, Chicago, Illinois, USA

* to whom correspondence should be addressed: jeffrey.hubbell@epfl.ch or jhubbell@uchicago.edu

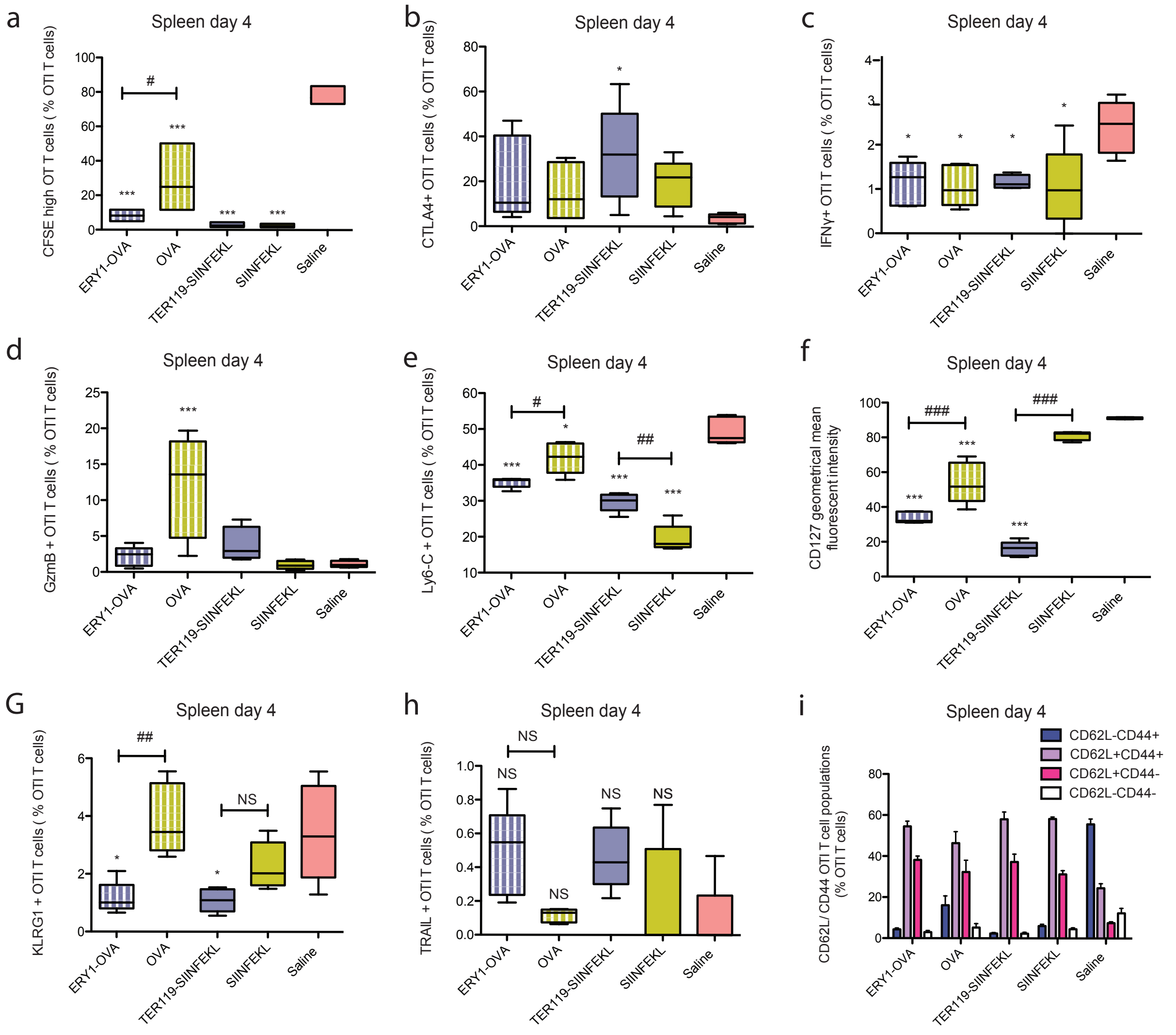

Supplementary figure S1: OTI T cell phenotypic markers expression in response to soluble and erythrocyte-bound antigens. *10^6 CFSE-labeled OTI CD8+ T cells were adoptively transferred on day 0 and mice treated with erythrocyte-bound, free antigen or saline the next day. Here, the full OVA protein was used with the ERY1-OVA antigen form, compared to free OVA; and only the CD8+ T cell epitope SIINFEKL was used with the TER119-SIINFEKL antigen form, compared with free SIINFEKL peptide. Spleens were collected on day 4 for flow cytometric analysis. (a) Proliferation of OTI T cells in response to soluble and erythrocyte-bound antigen. (b) CTLA4+, (c) IFNγ+, (d) GranzymeB+, (e) Ly6C+ OTI T cell populations. (f) CD127 expression, (g) KLRG1+, (h) TRAIL+, (i) CD44/CD62L OTI T cell populations in the spleen on day 4. Data represent mean ± SD of n=5. 1 way ANOVA *: respective to Saline group. *: < 0.05.*

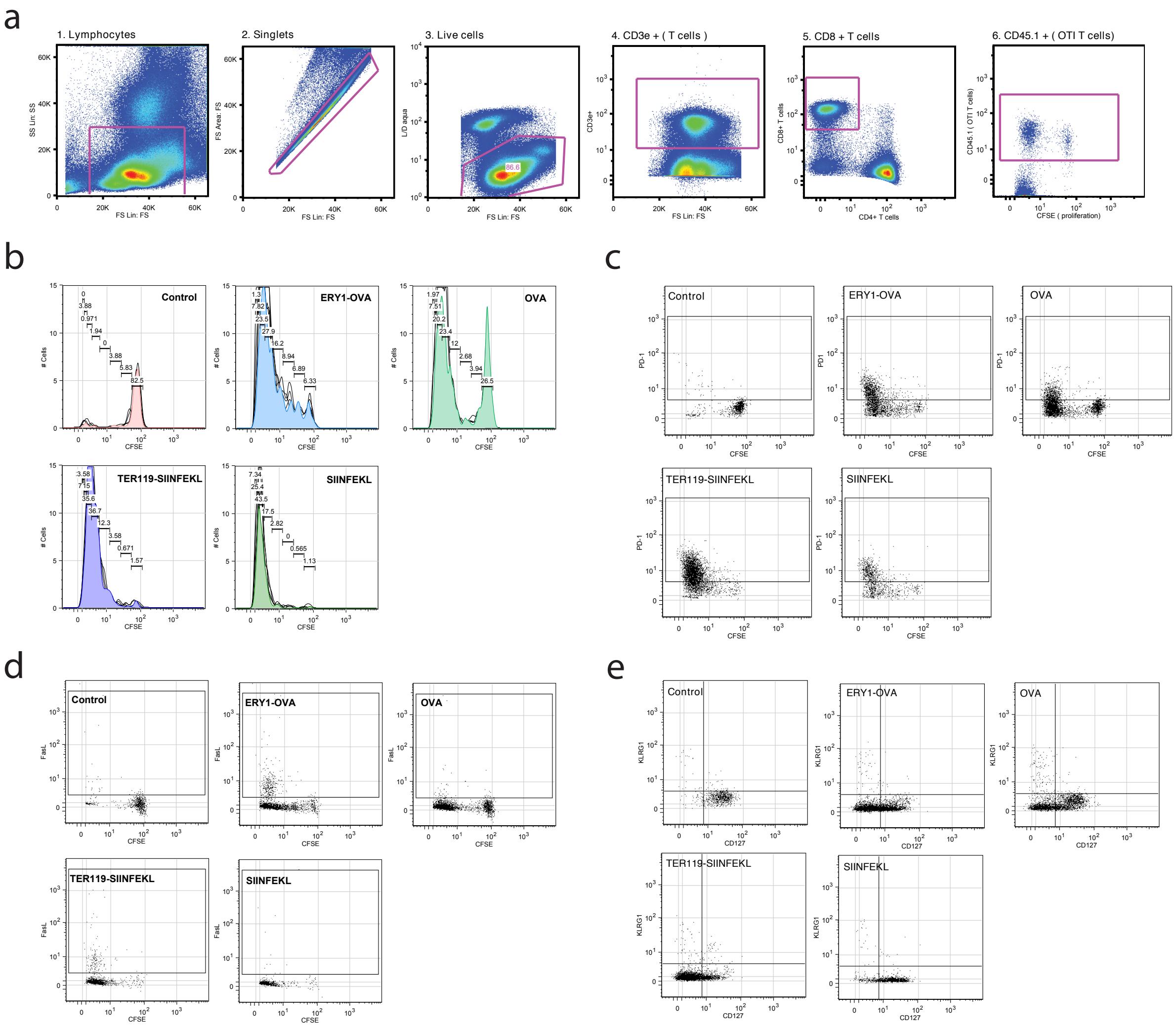

Supplementary figure S2: Gating strategies for selection of OTI CD8+ T cells and phenotypic markers. *(a) Representative plots are shown as they were used to identify OTI CD8+ T cells. FACS plots depict the gating strategy used to first isolate total cells based on forward scatter (FSC) and side scatter (SSC), and to exclude doublets using FS width versus area. OTI T cells were selected based on the expression of CD3e+, CD8+ and CD45.1+ markers. (b) FACS plots representing proliferation of OTI T cells and generation analysis. Gating strategies for (c) PD-1, (d) FasL and (e) KRLG1/CD127 markers. Data represent combined OTI T cell populations of n=5 animals.*

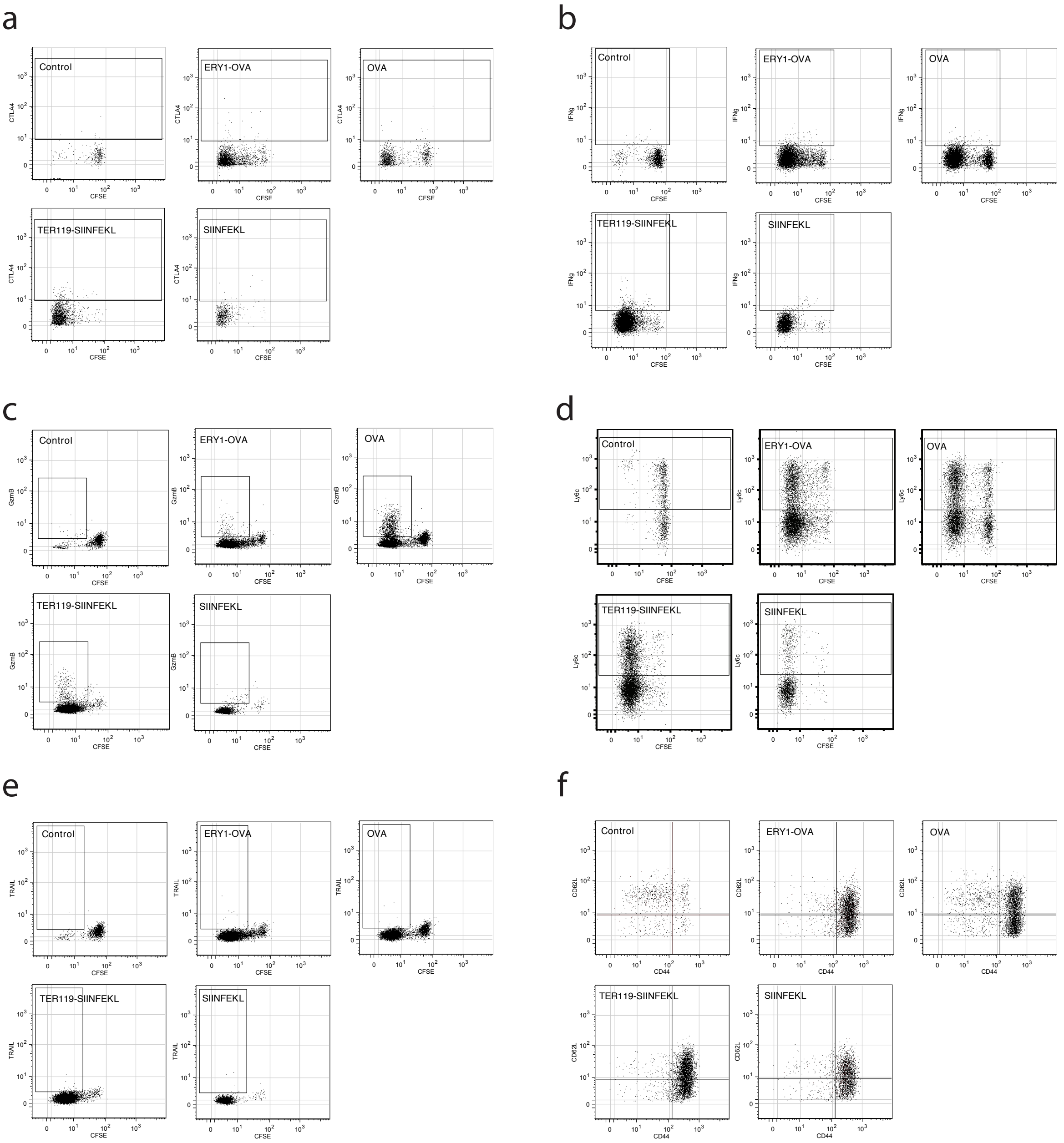

Supplementary figure S3: Gating strategies for phenotypic markers. *(a) CTLA4, (b) IFNγ, (c) GzmB, (d) Ly6c, (e) TRAIL, (f) CD62L/CD44 positive OTI T cell populations. Data represent combined OTI T cell populations of n=5 animals.*

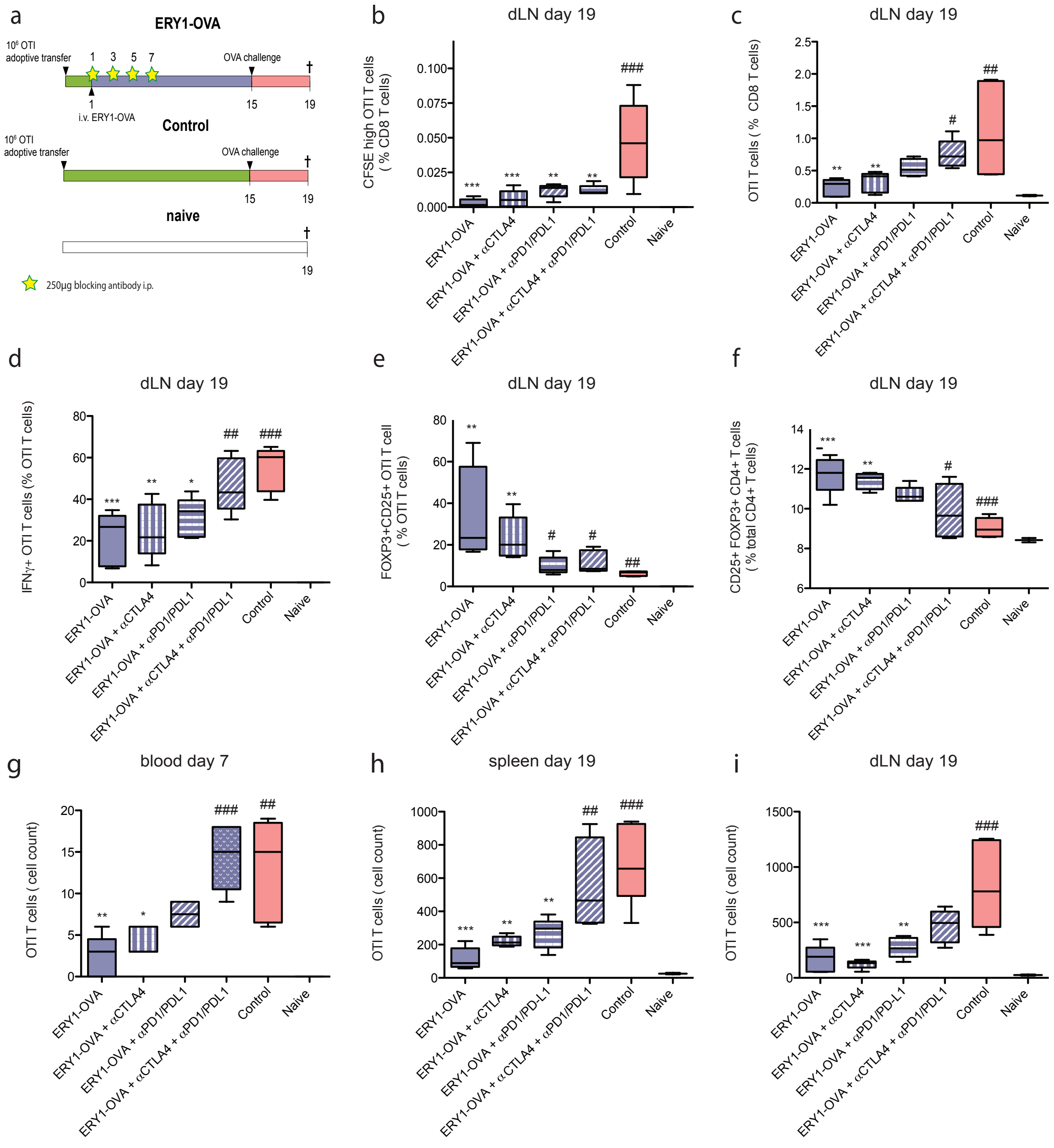

***Supplementary figure S4: Co-blockade of PD-1 and CTLA4 signaling abrogates antigen-specific T cell deletion by erythrocyte-targeted antigens**. (a) 10^6 OTI CFSE-labeled CD8+ T cells (CD45.1+) were adoptively transferred into C57BL/6 mice (CD45.2+). The next day, mice were treated with ERY1-OVA i.v. to induce proliferation and deletion of OTI T cells. 250 µg each of αPD-1 and αPD-L1 administered together, or 250 µg αCTLA4, or 250 µg each of all three antibodies were administered i.p. as indicated during the period of putative tolerization. Finally, mice were challenged i.d with OVA + LPS on day 15 and organs were harvested 4 days later for flow cytometric analysis. (b) Proliferation and (c) deletion of CFSE-labeled OTI T cells, measured in draining lymph nodes. (d) IFNγ+ OTI T cells from draining lymph nodes after 6 hour in vitro restimulation with SIINFEKL peptide. (e) CD25+FOXP3+ OTI T cell*

*and (f) endogenous CD25+FOXP3+ CD4+ T cell populations in draining lymph nodes on day 19. Absolute numbers of OTI T cells in (g) blood on day 7, (h) spleen and (i) draining lymph nodes on day 19. Data represent mean ± SD of n=5. 1 way ANOVA *: respective to Control group, #: respective to ERY1-OVA group. *, #: < 0.05, **, ##: < 0.01 , ***, ###: < 0.001.*

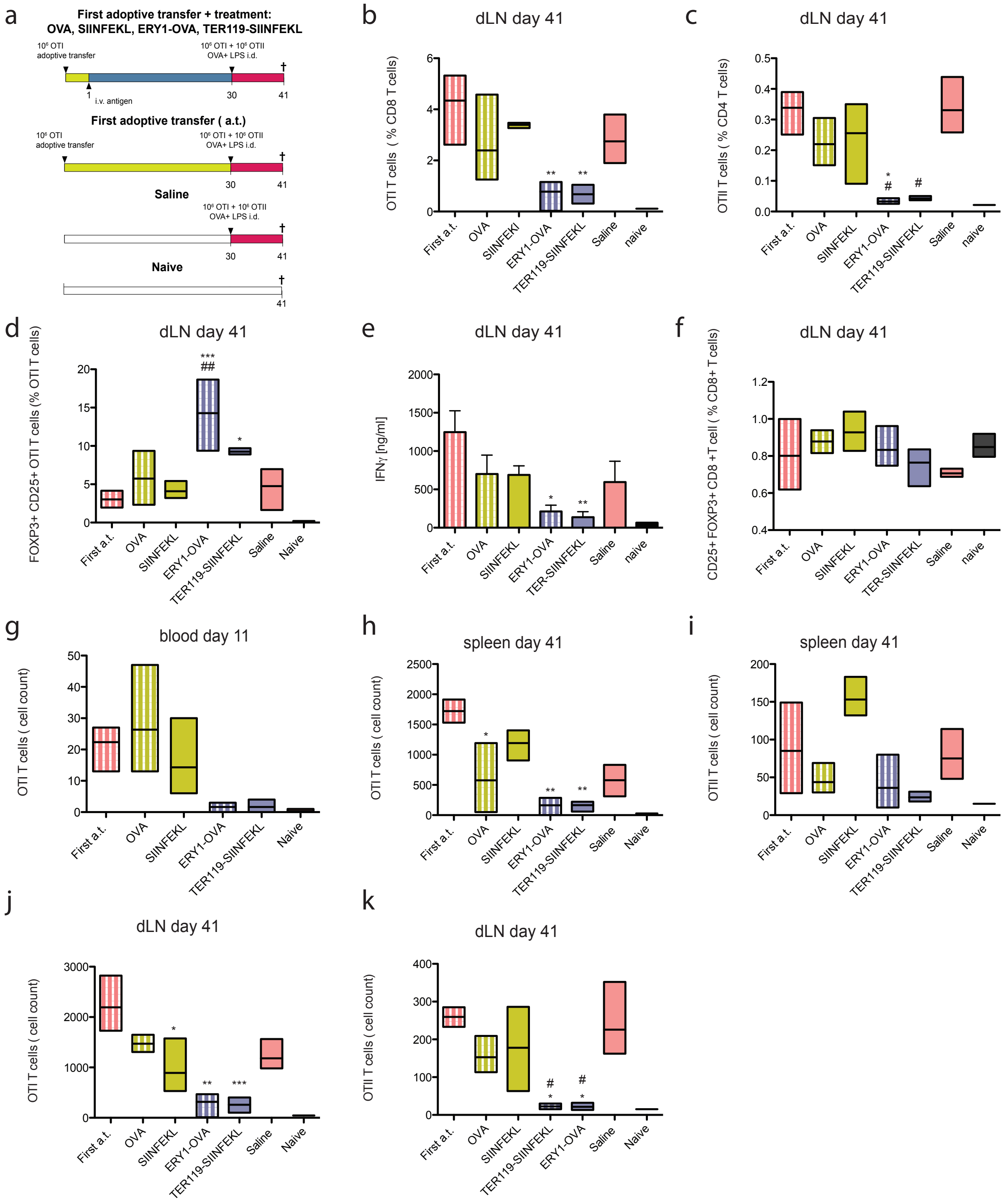

Supplementary figure S5: Memory of tolerance is induced after treatment with erythrocyte-targeted antigens. *(a) 10^6 OTI CD8+ CFSE-labeled T cells were adoptively transferred on day 0 and mice were treated with erythrocyte-bound, free antigen or saline the next day. One month later, a second adoptive transfer of OTI and OTII T cells, with OVA+LPS i.d. challenge rather than additional molecular tolerization, was performed to assess long-term memory of tolerance. (b) CD8+ OTI, (c) CD4+ OTII T cell and (d) CD25+FOXP3+ OTI T cells population in draining lymph nodes on day 41. (e) IFNγ production after 3 days in vitro restimulation of cells from draining lymph nodes with OVA,*

*measured by ELISA. (f) CD25+FOXP3+CD8+ T cell endogenous population in draining lymph nodes. Absolute numbers of (g) OTI CD8+ T cell in blood on day 11, (h) OTI CD8+ and (i) OTII CD4+ T cells in the spleen and (j) OTI CD8+ and (k) OTII CD4+ T cells in draining lymph nodes on day 41. Data represent mean ±SD of n=3. 1 way ANOVA *: respective to first a.t. group, # : respective to Saline group. *, #: < 0.05, **, ##: < 0.01, *** < 0.001.*

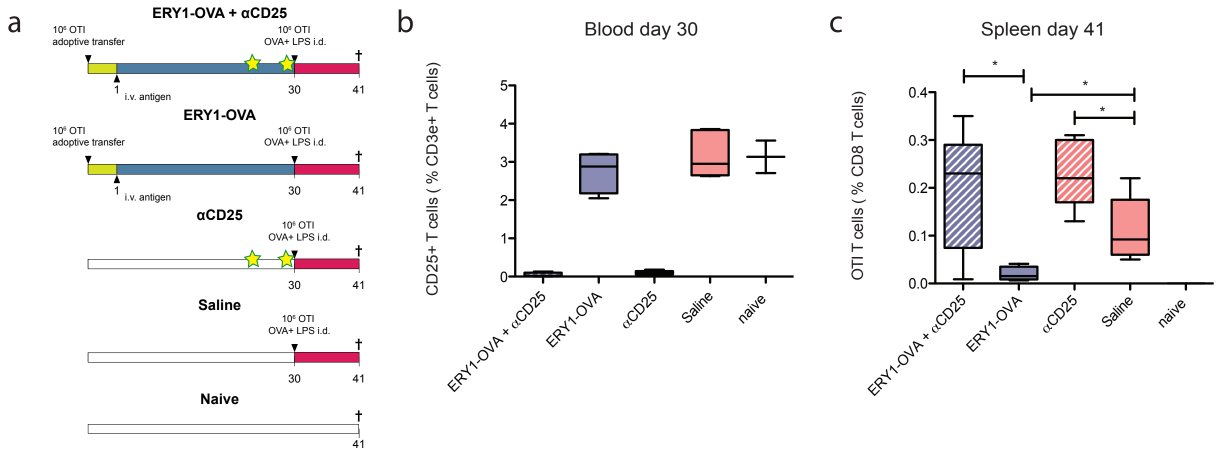

Supplementary figure S6: Depleting CD25+ T cells reverses memory of tolerance, but also affects homing of the OTI T cells after adoptive transfer. *(a) 10^6 OTI CD8+ T cell were adoptively transferred on day 0 and mice were treated with erythrocyte-bound OVA on day 1. To deplete CD25+ T cells, 250µg αCD25 blocking antibody was injected i.p. on day 23 and 30. One month after the first adoptive transfer, a second adoptive transfer of OTI with OVA+LPS i.d. challenge rather than additional molecular tolerization, was performed to assess long-term memory of tolerance. (b) CD25+ T cell population in blood on day 30. (c) OTI T cell population in the spleen on day 41. Data represent mean ±SD of n=5. 1 way ANOVA, *: < 0.05.*

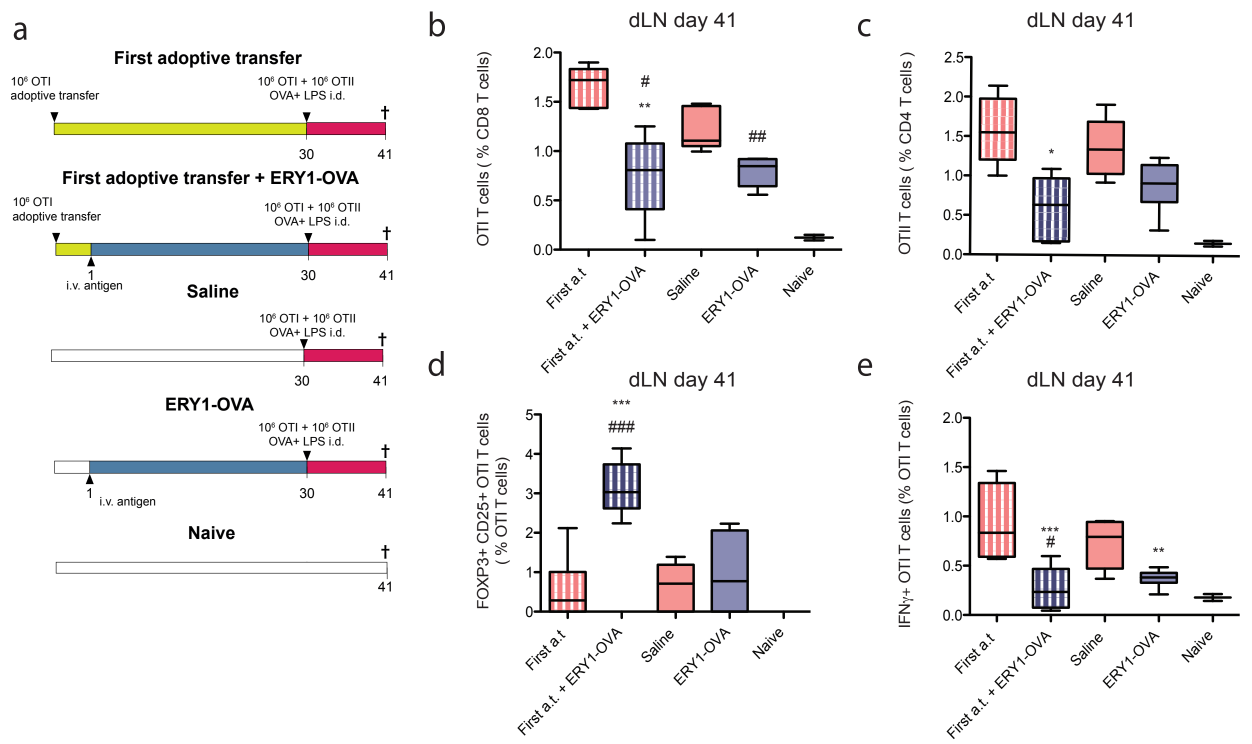

Supplementary figure S7: Memory of tolerance is not dependent on CD25+FOXP3+ OTI T cells but rather on endogenous regulation*. (a) 10^6 OTI CFSE-labeled CD8+ T cells were adoptively transferred into C57BL/6 mice. The next day, mice were treated i.v. with ERY1-OVA to induce proliferation and deletion of OTI T cells. One month later, a second adoptive transfer of OTI and OTII T cells, with OVA+LPS i.d. challenge rather than additional molecular tolerization, was performed to assess long-term memory of tolerance. (b) OTI CD8+ T cell, (c) OTII CD4+ T cell and (d) CD25+FOXP3+ OTI T cell populations in the draining lymph nodes on day 41. (e) IFNγ+ OTI T cells in draining lymph nodes after 6 hours in vitro restimulation with SIINFEKL. Data represent mean ±SD of n=5. 1 way ANOVA *: respective to first a.t. group, # respective to Saline group. *, #: < 0.05, **, ##: < 0.01, ***, ###<0.001.*

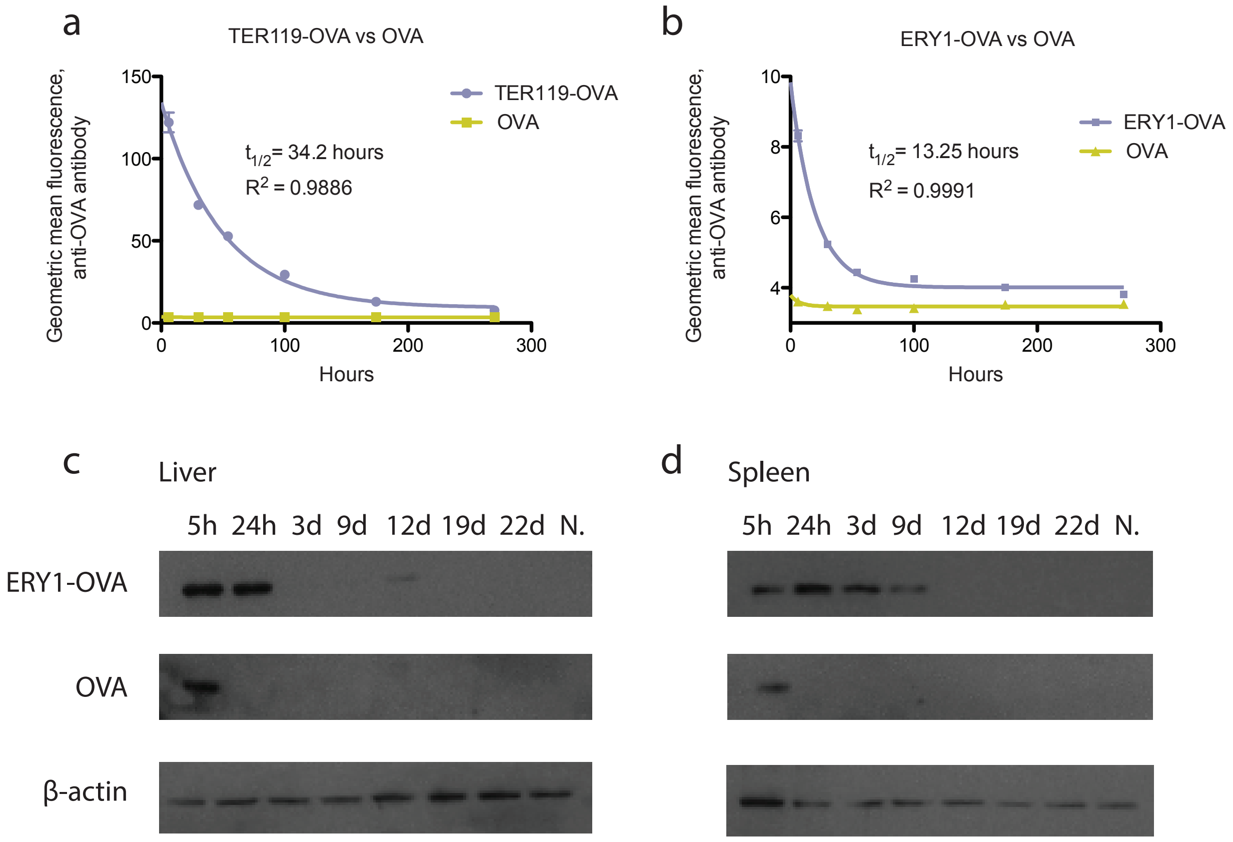

Supplementary figure S8: Half-life of erythrocyte-bound antigens in blood, spleen and liver*. (a) 223 pmol of OVA or TER119-OVA or (b) OVA or ERY1-OVA were administered i.v. and the circulation time of free or erythrocyte-bound OVA was determined by flow cytometry measurements of freshly-isolated blood at pre-determined time points using an anti-OVA antibody. Geometric mean fluorescence intensity of flow cytometry measurements. n = 2; one-phase exponential decay. (c) 223 pmol of OVA or ERY1-OVA, or saline were administered i.v. on day 0. Liver and (d) spleen were harvested at specific time ponits and protein extracts were used to determine clearance of the antigen by Western blotting using an anti-OVA antibody (exposure time: 15 min). β actin was used as assay control (exposure time : 10 sec).*
